# Immune-Enhancing Effects of Gwakhyangjeonggi-san in RAW 264.7 Macrophage Cells through the MAPK/NF-κB Signaling Pathways

**DOI:** 10.3390/ijms25179246

**Published:** 2024-08-26

**Authors:** Yun Hee Jeong, Hye Jin Yang, Wei Li, You-Chang Oh, Jang-Gi Choi

**Affiliations:** Korean Medicine (KM)-Application Center, Korea Institute of Oriental Medicine, 70, Cheomdanro, Dong-gu, Daegu 41062, Republic of Korea; runxi0333@kiom.re.kr (Y.H.J.); hjyang@kiom.re.kr (H.J.Y.); liwei1986@kiom.re.kr (W.L.)

**Keywords:** immunoregulation, macrophage, mitogen-activated protein kinase, nuclear factor-κB, immune function, Gwakhyangjeonggi-san

## Abstract

Gwakhyangjeonggi-san (GJS) is a traditional herbal medicine used in East Asia for the treatment of symptoms involving lower intestinal abnormalities; however, the effects of GJS on innate immunity and its cellular mechanisms of action have not been elucidated. In this study, we assessed the immune-enhancing activity and underlying mechanisms of GJS using RAW 264.7 murine macrophages. The results showed that GJS treatment significantly increased the secretion of nitric oxide and cytokines and their mRNA expression in macrophage RAW 264.7 cells without causing cytotoxicity. GJS treatment also significantly increased the production of reactive oxygen species, as well as inducing phagocytic activity, adhesion function, and migration ability, all of which improved the immune response. In addition, GJS activated nuclear factor-κB by promoting the phosphorylation and degradation of inhibitor of nuclear factor-κB alpha. Furthermore, GJS markedly increased the phosphorylation of mitogen-activated protein kinase in RAW 264.7 cells. These findings indicate that GJS has potential value as a dietary supplement for strengthening immunity.

## 1. Introduction

The immune response is an essential process that includes innate and adaptive immunity. It is the first line of defense against pathogenic microbial and viral infections [1]. Immune function dysregulation can affect the development and progression of various diseases, including cardiovascular disease, diabetes, cancer, autoimmune diseases, and infectious diseases [2,3]. Therefore, the regulation of the immune response is important for maintaining health by preventing diseases. Innate immunity acts as the first line of defense against a variety of foreign pathogens. Macrophages are professional phagocytes of the innate immune system that participate in the detection, phagocytosis, and destruction of bacteria and other harmful organisms [4].

Macrophages regulate the immune response by secreting nitric oxide (NO), reactive oxygen species (ROS), and cytokines, such as tumor necrosis factor (TNF)-α, interleukin (IL)-6, and IL-1β [5]. They play an important role in linking innate and adaptive immunity by activating natural killer cells, T cells, and B cells to initiate adaptive immune responses [6]. Therefore, macrophages are specialized cells that have an important role in mechanisms associated with the immune system. Thus, not only are macrophages used in the study of natural products that can enhance the immune system, but their activation is considered a target for immunomodulation [7,8,9].

Gwakhyangjeonggi-san (GJS) is a traditional herbal medicine that contains medicinal herbs such as Agastachis Herba, Perillae Folium, Angelicae Dahuricae Radix, and Arecae Pericarpium. It is used to treat diarrhea, lower abdominal pain, and lower intestinal disorders [10] and has recently been reported to exert therapeutic effects on allergies, respiratory and cardiovascular diseases, and bacterial infections [11,12,13,14]; however, the effects of GJS on macrophage-mediated regulation of immune function and its underlying molecular mechanisms have not been established. Therefore, in this study, we determined the effects of treating murine macrophage RAW 264.7 with GJS on the activation of immune function and its underlying mechanism. Also, the individual components of GJS were identified through ultra high-performance liquid chromatography (UHPLC)-UV-MS/MS-based phytochemical analysis.

## 2. Results

### 2.1. GJS Induces NO Secretion in Macrophages without Cytotoxicity

**Figure 1:**
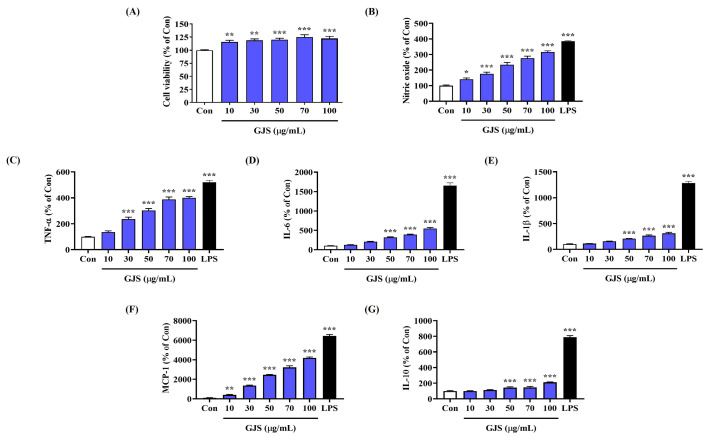
Effects of GJS on (**A**) cell viability and secretion of (**B**) NO and (**C**–**G**) cytokines in RAW 264.7 cells. Cells were incubated with 10–100 μg/mL GJS or 10 ng/mL LPS. Control cells were incubated with vehicle alone. Data are presented as mean ± standard error of the mean of nine independent experiments. Statistical significance was defined as * *p* < 0.05, ** *p* < 0.01, and *** *p* < 0.001 (vs. control).

Prior to the experiments, the influence of GJS on RAW 264.7 cell viability was assessed using a cell-counting kit (CCK) assay, which indicated no cytotoxicity up to a concentration of 100 μg/mL (Figure 1A). Therefore, subsequent experiments were carried out using 100 μg/mL GJS or less.

Activated macrophages secrete NO, which can enhance innate immunity. Therefore, to determine the effects of GJS on macrophage activation, we examined whether it induced NO secretion in RAW 264.7 cells, by measuring nitrite concentration in the culture medium using the Griess reagent. GJS treatment significantly induced NO secretion in macrophages in a concentration-dependent manner (Figure 1B). Lipopolysaccharide (LPS, 10 ng/mL) was used as a positive control, which showed excellent immune response activation by inducing NO secretion at a high level.

### 2.2. GJS Induces Cytokine Secretion in Macrophages and Modulates Gene Expression

To further determine the effects of GJS on macrophage activation through induction of NO secretion, the production of several cytokines related to immune response activation was measured by enzyme-linked immunosorbent assay (ELISA). When RAW 264.7 cells were treated with GJS at various concentrations, the production of TNF-α, IL-6, IL-1β, monocyte chemoattractant protein (MCP)-1, and IL-10 was significantly increased (Figure 1C–G). Most assays showed statistical significance following treatment with GJS above 50 μg/mL.

**Figure 2:**
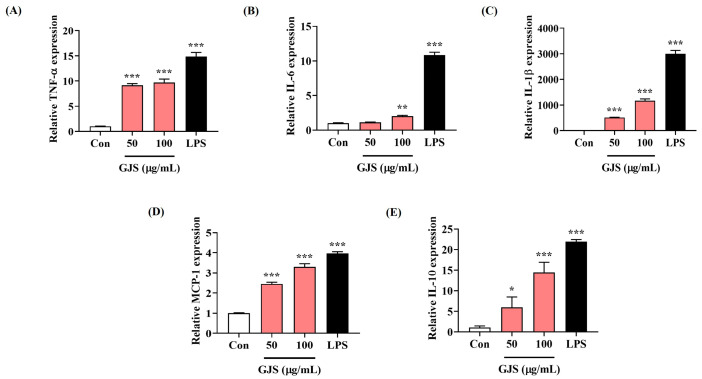
Effect of GJS on (**A**–**E**) the expression of cytokine mRNAs in RAW 264.7 cells. Cells were incubated with 50 or 100 μg/mL GJS or 10 ng/mL LPS. Control cells were incubated with vehicle alone. Data are presented as mean ± standard error of the mean of nine independent experiments. Statistical significance was defined as * *p* < 0.05, ** *p* < 0.01, and *** *p* < 0.001 (vs. control).

Next, we determined the effects of GJS on cytokine mRNA expression, using quantitative polymerase chain reaction (qPCR). GJS significantly induced cytokine mRNA expression in macrophages, as did cytokine secretion (Figure 2). LPS treatment also exhibited strong induction of cytokine production and gene expression.

### 2.3. GJS Treatment Significantly Increases Intracellular ROS Accumulation in Macrophages

**Figure 3:**
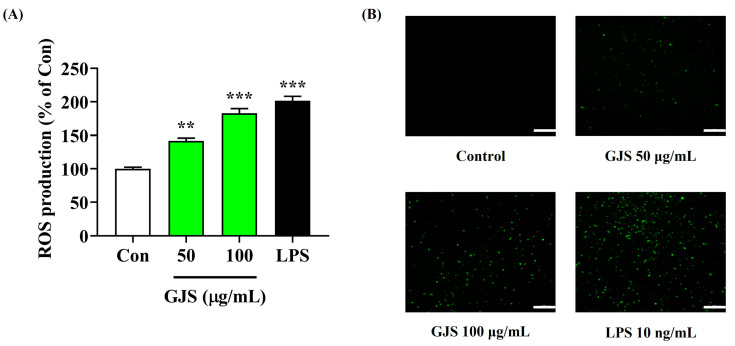
Effects of GJS on intracellular ROS production in RAW 264.7 cells. (**A**,**B**) Cells were incubated with 50 or 100 μg/mL GJS or 10 ng/mL LPS. Control cells were incubated with vehicle alone. (**B**) Scale bar = 200 μm. (**A**) Data are presented as mean ± standard error of the mean of nine independent experiments. Statistical significance was defined as ** *p* < 0.01 and *** *p* < 0.001 (vs. control).

Because ROS is an important factor that enables macrophages to engage in smooth phagocytosis and plays an important role in immune defense and regulation, we measured ROS accumulation in RAW 264.7 cells using the H_2_DCFDA fluorescence assay. GJS significantly increased ROS accumulation in macrophages in a concentration-dependent manner (Figure 3). Moreover, treatment with 100 μg/mL GJS resulted in a similar level of ROS generation compared with LPS as the positive control.

### 2.4. Strengthening Effects of GJS on the Immune Function of Macrophages

**Figure 4:**
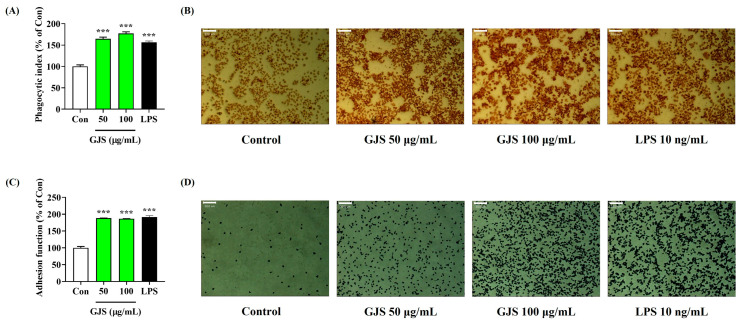
Effects of GJS on (**A**,**B**) phagocytic activity and (**C**,**D**) the adhesion function of RAW 264.7 cells. Cells were incubated with 50 or 100 μg/mL GJS or 10 ng/mL LPS. Control cells were incubated with vehicle alone. (**B**,**D**) Scale bar = 50 μm. (**A**,**C**) Data are presented as mean ± standard error of the mean of nine independent experiments. Statistical significance was defined as *** *p* < 0.001 (vs. control).

**Figure 5:**
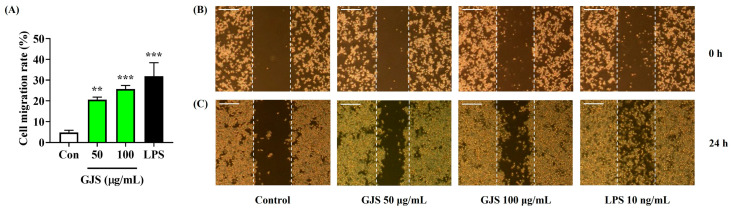
Effects of GJS on the migration of RAW 264.7 cells. (**A**–**C**) Cells were incubated with 50 or 100 μg/mL GJS or 10 ng/mL LPS. Control cells were incubated with vehicle alone. (**B**,**C**) Scale bar = 100 μm. (**A**) Data are presented as mean ± standard error of the mean of nine independent experiments. Statistical significance was defined as ** *p* < 0.01 and *** *p* < 0.001 (vs. control).

To further examine the effects of GJS on enhancing the immune function of macrophages, several tests related to macrophage activation were performed. Because phagocytosis is one of the main functions of macrophages to enhance immune function, we evaluated the effects of GJS on phagocytosis and adhesion in RAW 264.7 cells. Phagocytic function assessed using neutral red showed that GJS had a positive effect on enhancing the immune function of macrophages (Figure 4A). We also confirmed that GJS treatment significantly activated the adhesion function of RAW 264.7 cells (Figure 4B). Furthermore, the efficacy of GJS was analyzed using a wound healing assay, in which GJS treatment significantly improved the proliferation and migration activity of the macrophages (Figure 5). Based on these results, we confirmed that GJS improved the immune function of macrophages by promoting phagocytic activity, adhesion, proliferation, and migration.

### 2.5. GJS Activates the Mitogen-Activated Protein Kinase (MAPK) and Nuclear Factor (NF)-κB Signaling Pathways in Macrophages

**Figure 6:**
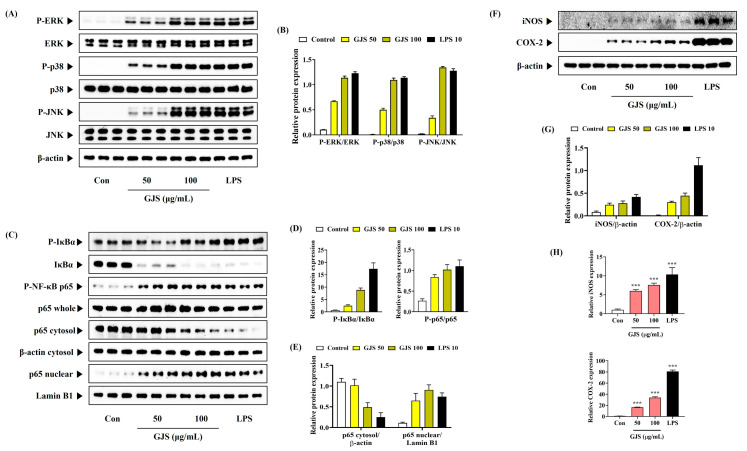
Effects of GJS on (**A**,**B**) the phosphorylation of MAPK, (**C**–**E**) activation of NF-κB, and (**F**–**H**) expression of iNOS and COX-2 in RAW 264.7 cells. (**A**–**H**) Cells were incubated with 50 or 100 μg/mL GJS or 10 ng/mL LPS. Control cells were incubated with vehicle alone. (**B**,**D**,**E**,**G**) The histogram graphs show protein expression relative to those of a housekeeping protein. (**B**,**D**,**E**,**G**) Data are presented as analysis values from Western blot experiment using three independently obtained protein samples. (**H**) Data are presented as mean ± standard error of the mean of nine independent qPCR experiments. Statistical significance was defined as *** *p* < 0.001 (vs. Control).

To identify the signaling pathways involved in the effects of GJS on the immune function of macrophages, the activation of the NF-κB and MAPK signaling pathways was assessed by Western blot analysis. GJS treatment increased the phosphorylation of extracellular signal-regulated kinases (ERK), p38, and c-Jun N-terminal kinases (JNK) MAPK in RAW 264.7 cells in a concentration-dependent manner (Figure 6A). Similar to MAPK activation, GJS also activated the NF-κB signaling pathway. It promoted the degradation and phosphorylation of inhibitor of nuclear factor-κB alpha (IκBα), an inhibitory protein of NF-κB, and showed an effect on its translocation to the nucleus through increased p65 phosphorylation (Figure 6B). Based on its effects on the expression of these proteins, GJS appears to have strengthened the immune function of the macrophages by activating the MAPK and NF-κB pathways.

### 2.6. GJS Increases Inducible Nitric Oxide Synthase (iNOS) and Cyclooxygenase (COX)-2 Enzyme Expression in Macrophages

Because iNOS and COX-2 play an important role in immune activation by catalyzing the production of NO and prostaglandin (PG)E_2_, respectively, we determined the effects of GJS on the expression of iNOS and COX-2 protein and mRNA in RAW 264.7 cells by Western blot analysis and qPCR. iNOS and COX-2 protein and mRNA expression were upregulated by GJS in a concentration-dependent manner (Figure 6C).

### 2.7. UHPLC Coupled with Quadrupole-Orbitrap Mass Spectrometry (UHPLC-UV-HRMS) Analysis of Phytochemicals in GJS

**Figure 7:**
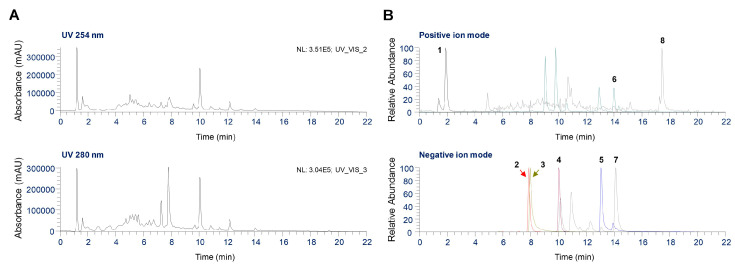
UHPLC-UV-HRMS analysis of eight components in the GJS. (**A**) UV chromatograms of GJS at wavelengths of 254 nm and 280 nm. (**B**) The EIC for eight identified components in positive and negative ion modes.

Phytochemical analysis of GJS was performed using UHPLC-UV-MS/MS. Eight major components were identified, which included acacetin from *A. rugosa*, rosmarinic acid from *P. frutescens*, oxypeucedanin from *A. dahurica*, arecoline hydrobromide from *A. catechu*, atractylenolide II from *A. japonica*, hesperidin from *C. unshiu*, platycodin D from *p. grandiflorum*, and glycyrrhizin from *G. uralensis* [15,16,17,18]. Both positive and negative ion modes were used to acquire full MS and MS2 spectra for each analyte. Figure 7A presents UV chromatograms of GJS at wavelengths of 254 nm and 280 nm. Figure 7B shows the extracted ion chromatograms (EIC) for the precursor ion *m*/*z* values of each analyte. Table 1 shows the eight components identified in GJS along with their retention times, measured precursor ions, and MS2 fragments compared with those of the corresponding standards. Flavonoids, saponins, and phenolic compounds were ionized in negative ion mode, whereas alkaloids, coumarin, and terpenoids were detected in positive ion mode.

## 3. Discussion

Innate immunity is the body’s first line of defense against various external pathogens. Macrophages are professional phagocytes of the innate immune system that detect and destroy harmful organisms such as bacteria and viruses [1,4]. They also play a role in initiating the adaptive immune response by presenting antigens to various immune cells [6]. Therefore, the activation of macrophages is closely related to increased immune function. GJS is an important traditional herbal decoction that has been traditionally used in East Asia. A previous study reported that GJS exerts positive effects on allergies, respiratory and cardiovascular diseases, and bacterial infections [11,12,13,14]; however, the immune-enhancing effect of GJS on murine macrophage RAW 264.7 cells and its molecular mechanisms were previously unknown. Therefore, we examined the macrophage-mediated immune-enhancing efficacy of GJS and its underlying mechanism.

NO is a gaseous free radical that acts as an immunoregulatory mediator involved in both the innate and adaptive immune response [19]. Increased NO release is associated with the upregulation of iNOS levels and it enhances immunomodulatory activity [20]. NO is positively associated with the phagocytic effect of macrophages [21]. Our results indicated that GJS significantly increased NO secretion and markedly elevated iNOS expression in a concentration-dependent manner. COX-2 synthesizes PGE_2_ from arachidonic acid and plays an important role in immune regulation [22]. GJS markedly increases both the protein and mRNA expression of COX-2 in a dose-dependent manner. In addition, several cytokines associated with immune function activation contribute to regulating the immune system of macrophages [23]. In the present study, we showed that GJS increased TNF-α, IL-6, IL-1β, MCP-1, and IL-10 cytokine levels and their mRNA expression in RAW 264.7 cells. This suggests that GJS has an immune-enhancing function by upregulating NO, iNOS, COX-2, and cytokine production.

Phagocytosis is an important biological process that enables the host to protect itself from foreign particles and microorganisms. When macrophages are activated, they initiate phagocytosis to remove debris and other foreign substances through the innate immune response. Increased phagocytic activity is a typical manifestation of macrophage activation [24]. ROS is an important factor in destruction of pathogens by phagocytes and plays a central role in immune defense and regulation [25]. Therefore, we examined the effects of GJS on phagocytosis and ROS production in RAW 264.7 cells. The results indicated that GJS effectively promoted phagocytosis and ROS production in RAW264.7 cells. To determine the additional immune-enhancing functions of GJS, we evaluated their effects on the adhesion and migration abilities of RAW 264.7 cells. GJS treatment significantly improved the adhesion and migration activity of macrophages compared with untreated control cells. These data suggest that GJS strengthens immune function and eliminates foreign pathogens more efficiently by activating macrophages.

MAPK and NF-κB are important signaling pathways that regulate the immune response [26]. MAPKs are serine–threonine kinases consisting of three major subgroups: ERK, p38, and JNK. MAPK phosphorylation induces the activation of the NF-κB pathway and promotes the production of immunoregulatory mediators such as NO, iNOS, COX-2, TNF-α, IL-6, and IL-1β in macrophages [27]. Therefore, we investigated whether GJS treatment increased the activation of MAPK and found that GJS induced the phosphorylation of ERK, p38, and JNK in a concentration-dependent manner. NF-κB is a ubiquitous transcription factor composed of p50 and p65 subunits. Activation of the NF-κB pathway results in IκBα phosphorylation and degradation, followed by the translocation of the p65 subunit of NF-κB to the nucleus [28]. Western blot analysis revealed that GJS treatment significantly increased the nuclear translocation of p65 by increasing the phosphorylation and degradation of IκBα in a concentration-dependent manner. These findings indicate that GJS enhances the immune response via stimulating macrophages through activating the MAPK and NF-κB signaling pathways.

We performed phytochemical analyses using UHPLC-UV-HRMS to determine the relationships between the immune-enhancing activity of GJS and its components. Eight major compounds were identified, including arecoline hydrobromide, hesperidin, rosmarinic acid, platycodin D, glycyrrhizin, oxypeucedanin, acacetin, and atractylenolide II. Based on these results, we determined whether the immune-enhancing effects of GJS were related to its constituent herbs and components. Previous studies have demonstrated that *Platycodon grandiflorum* induces macrophage proliferation and exhibits immune-enhancing effects through NF-κB and AMP-activated kinase activation [29]. In addition, the components of *Citrus unshiu* fruit peel were shown to activate MAPK and NF-κB in RAW 264.7 cells, induce the production of IL-6, TNF-α, and NO, and stimulate macrophages [30]. Furthermore, when *Glycyrrhiza uralensis* was used as a feed supplement for yellow catfish, toll-like receptor-NF-κB signaling was activated and IL-1β and IL-8 cytokine secretion increased, thereby enhancing resistance to infection [31]. Platycodin D, a major component of *Platycodon grandiflorum* roots and a component of GJS, significantly inhibits tumor growth by increasing interferon-γ, TNF-α, IL-6, and IL-2 levels and improves immune function [32]. Therefore, the immune-enhancing effects of GJS may occur, in part, through the presence of *Citrus unshiu*, *Glycyrrhiza uralensis*, *Platycodon grandiflorum*, and its main component platycodin D.

## 4. Materials and Methods

### 4.1. Materials and Reagents

Dulbecco’s modified Eagle’s medium (DMEM), fetal bovine serum (FBS), and antibiotics were purchased from Hyclone (Logan, UT, USA). A CCK was obtained from Dojindo Molecular Technologies, Inc. (Kumamoto, Japan). ELISA kits for cytokine detection were obtained from Invitrogen (Waltham, MA, USA). The RNA extraction kit was purchased from iNtRON (Sungnam, Korea). DNA synthesizing kits, oligonucleotide primers, and AccuPower 2X Greenstar qPCR Master Mix (ROX) were obtained from Bioneer (Daejeon, Korea). Various primary and horseradish peroxidase (HRP)-conjugated secondary antibodies for Western blot analysis were acquired from Cell Signaling Technology, Inc. (Boston, MA, USA). The polyvinylidene difluoride (PVDF) membrane was purchased from Millipore (Bedford, MA, USA).

### 4.2. Preparation of GJS

GJS consists of 13 medicinal herbs (Table 2), all of which were acquired from Humanherb (Daegu, Korea). The herbs were authenticated by Dr. Wei Li of the Korea Institute of Oriental Medicine (Daegu, Korea). All voucher specimens were deposited in the herbal bank at the Korean Medicine Application Center, Korea Institute of Oriental Medicine (voucher number: C24-12). The herbal mixture (32.0 g) was extracted with 1 L of hot water for 3 h. The extract was sieve-filtered and freeze-dried. The resulting GJS powder was kept in a desiccator at −20 °C before use.

### 4.3. Cell Culture

Murine macrophage-like RAW 264.7 cells were obtained from the American Type Culture Collection (Manassas, VA, USA). RAW 264.7 cells were cultured in DMEM supplemented with penicillin (100 U/mL), streptomycin (100 μg/mL), and 10% heat-inactivated FBS at 37 °C in a humidified incubator containing 5% CO_2_.

### 4.4. Cell Viability Test

Cell viability was measured using the CCK assay following the manufacturer’s protocol. Cells were seeded into 96-well culture plates (5 × 10^4^ cells/well) and incubated with GJS for 24 h. CCK solution (10 μL) was added and incubated for an additional 1 h. The optical density was measured at 450 nm using a microplate reader (SpectraMax i3; Molecular Devices, San Jose, CA, USA).

### 4.5. NO Production

The Griess reaction was used to measure NO production in macrophages. Cells were seeded in 96-well culture plates (5 × 10^4^ cells/well) and were incubated with GJS or LPS for 24 h. LPS (10 ng/mL) was used as a positive control. Griess reagent (1% sulfanilamide, 0.1% N-1-napthylethylenediamine dihydrochloride, and 2.5% phosphoric acid) was mixed with an equal volume of cell supernatant and incubated at room temperature (RT) for 5 min [33]. The absorbance was read at 570 nm using a microplate reader. The quantity of nitrite in the samples was determined using sodium nitrite as a standard.

### 4.6. Determination of Cytokines

The secretion of cytokines was assessed using ELISA kits following the manufacturer’s instructions. Cells were grown in 24-well culture plates (2.5 × 10^5^ cells/well) and treated with GJS or LPS for 24 h. The culture medium was centrifuged at 15,000× *g* for 10 min and the supernatants were collected. Cytokine levels were measured at 450 nm using a microplate reader.

### 4.7. RNA Extraction, DNA Synthesis, and qPCR

Total RNA was isolated from RAW 264.7 cells using easy-BLUE™ RNA reagent. Total RNA (1 μg) was reverse transcribed into cDNA using RevoScript™ RT PreMix. AccuPower^®^ 2X Greenstar qPCR Master Mix was used for amplification, according to the manufacturer’s instructions. The primer sequences for qPCR are listed in Table 3. Amplification and analysis were performed using a QuantStudio 6 Flex Real-time PCR System (Thermo Scientific, Rockford, IL, USA). The samples were compared using the relative Ct method. The relative expression of the target genes was normalized to β-actin as an endogenous control.

### 4.8. Measurement of Intracellular ROS Levels

Intracellular ROS generation was measured using a fluorescent probe, H_2_DCFDA. Cells were seeded in 96-well culture plates (5 × 10^4^ cells/well) and incubated with GJS or LPS. After 6 h of GJS or LPS treatment, the cells were stained with 15 μM of H_2_DCFDA for 30 min at 37 °C in the dark. The stained cells were washed twice with ice-cold phosphate-buffered saline (PBS). Fluorescence was measured using a microplate reader at an excitation wavelength of 485 nm and an emission wavelength of 525 nm. Representative fluorescence images of the cells were captured under an inverted fluorescence microscope (Eclipse Ti, Nikon, Tokyo, Japan).

### 4.9. Evaluation of Phagocytosis

The phagocytic activity of RAW 264.7 cells was assessed using the neutral red method [34]. Cells were seeded into 96-well culture plates (1 × 10^4^ cells/well) and incubated with GJS or LPS for 24 h. The culture medium was discarded and 0.1% neutral red solution was added to each well and incubated for 1 h. The cells were subsequently washed three times with PBS and incubated with cell lysis buffer (1% glacial acetic acid: ethanol = 1:1) at RT for 2 h. The fluorescence intensity was measured at 540 nm using a microplate reader. The phagocytic index was calculated as described previously [34].

### 4.10. Measurement of Adhesion Function

Cells were seeded into 6-well culture plates (1 × 10^6^ cells/well) and incubated with GJS or LPS for 24 h. After incubation, the cells0 were collected by scraping, centrifuged, seeded into 24-well culture plates at a density of 2.5 × 10^5^ cells/well, and incubated for 1 h. The cells were then washed twice with PBS, fixed with 4% paraformaldehyde for 15 min, and washed twice with PBS. The cells were stained with 0.2% crystal violet solution for 15 min [34], washed twice with PBS, and photographed at 100× magnification.

### 4.11. Measurement of Migration Activity

Cells were seeded into 12-well culture plates (5 × 10^5^ cells/well) and incubated for 24 h. After the cells adhered to the wall, a scratch was introduced at the bottom of the culture plate with a 200 μL pipette tip. The cells were incubated with GJS or LPS for 24 h. The scratches were observed under an inverted microscope at 0 and 24 h, and the migration rate was calculated using Image J software (Version 1.53e, National Institutes of Health, Bethesda, MD, USA).

### 4.12. Western Blot Analysis

Cultured RAW 264.7 cells were washed with ice-cold PBS and lysed with radioimmunoprecipitation assay lysis buffer (Millipore, Bedford, MA, USA) supplemented with a protease and phosphatase inhibitor cocktail. Nuclear and cytoplasmic proteins were fractionated using NE-PER™ Nuclear and Cytoplasmic Extraction Reagents (Thermo Scientific) according to the manufacturer’s instructions. Protein samples were subjected to sodium dodecyl sulfate-polyacrylamide gel electrophoresis and transferred to PVDF membranes. The membranes were blocked with 3% bovine serum albumin at RT for 1 h followed by incubation with primary antibody overnight at 4 °C. The membranes were washed with Tris-buffered saline containing 0.1% Tween 20 and incubated with HRP-conjugated secondary antibodies. The protein bands were detected using Clarity™ Western ECL Substrate (Bio-Rad, Hercules, CA, USA), and relative protein expression was visualized using the ChemiDoc imaging system (Bio-Rad). Band intensity was analyzed using Image J software with control value normalization. Table 4 lists the primary and secondary antibodies.

### 4.13. UHPLC-UV-HRMS Analysis

UHPLC-UV-HRMS (Thermo Fisher Scientific, San Jose, CA, USA) was used to identify the phytochemicals in the GJS. Detailed analytical conditions and methods were as described previously [35]. Briefly, chromatographic separation was achieved using a gradient elution with 0.1% formic acid in water (solvent A, *v*/*v*) and acetonitrile (solvent B) on an Acquity BEH C18 analytical column (100 × 2.1 mm, 1.7 μm). Data acquisition and analysis were carried out using Xcalibur v.4.2 and Tracefinder v.4.0 software (Thermo Fisher Scientific, Foster, CA, USA). The reference standards for the analysis were obtained from Sigma-Aldrich Co. (St. Louis, MO, USA), Targetmol (Wellesley Hills, MA, USA), and ChemFaces (Wuhan, China), all with purity exceeding 97%.

### 4.14. Statistical Analysis

GraphPad Prism (version 8.0) software was used for statistical analysis. The data are expressed as means ± standard error of the mean for all experiments, and all quantitative data were representative of nine independent experiments. Statistical significance was analyzed by one-way analysis of variance with Dunnett’s test after comparing the control and each treated sample. * *p* values of <0.05, ** < 0.01, and *** < 0.001 (vs. control) were considered statistically significant.

## 5. Conclusions

This study demonstrated that GJS has strong immune activation effects. It increases the production of NO, cytokines, and ROS, and activates phagocytosis, promotes adhesion, and enhances migration in macrophage RAW 264.7 cells. The immune-enhancing effects of GJS are exerted through the activation of the MAPK and NF-κB signaling pathways. Moreover, the constituent herbs and components of GJS, including *Citrus unshiu*, *Glycyrrhiza uralensis*, *Platycodon grandiflorum*, and platycodin D, may be closely related to its immune-enhancing effects. These results suggest that GJS has potential value as an adjuvant to enhance immunity.

## Figures and Tables

**Table 1:** Identification of phytochemicals in GJS using UHPLC-UV-HRMS.

No	*t*_R_(min)	Chemical formula	Adduct	Estimated (*m*/*z*)	Calculated (*m*/*z*)	Error (ppm)	Identification	Source
1	1.85	C_8_H_13_NO_2_	[M+H]^+^	156.1019	156.1021	0.9019	Arecoline hydrobromide	*A. catechu*
2	7.88	C_28_H_34_O_15_	[M−H]^−^	609.1825	609.1830	0.9101	Hesperidin	*C. unshiu*
3	7.97	C_18_H_16_O_8_	[M−H]^−^	359.0772	359.0779	1.6998	Rosmarinic acid	*P. frutescens*
4	10.07	C_57_H_92_O_28_	[M−H]^−^	1223.5702	1223.5704	0.1672	Platycodin D	*P.grandiflorum*
5	13.07	C_42_H_62_O_16_	[M−H]^−^	821.3965	821.3976	1.3063	Glycyrrhizin	*G. uralensis*
6	13.99	C_16_H_14_O_5_	[M+H]^+^	287.0914	287.0916	0.6383	Oxypeucedanin	*A. dahurica*
7	14.11	C_16_H_12_O_5_	[M−H]^−^	283.0612	283.0614	0.6036	Acacetin	*A. rugosa*
8	17.43	C_15_H_20_O_2_	[M+H]^+^	233.1536	233.1538	0.9827	Atractylenolide II	*A. japonica*

**Table 2:** The herbal ingredients and composition ratio of GJS.

Scientific Name of Herbs	Medicinal Parts	Composition Ratio (%)
*Agastache rugosa*	Herba	18.75
*Perilla frutescens*	Leaf	12.5
*Angelica dahurica*	Root	6.25
*Areca catechu*	Peel of fruit	6.25
*Poria cocos*	Sclerotium	6.25
*Magnolia officinalis*	Bark	6.25
*Atractylodes japonica*	Rhizome	6.25
*Citrus unshiu*	Peel of fruit	6.25
*Pinellia ternata*	Tuber	6.25
*Platycodon grandiflorum*	Root	6.25
*Glycyrrhiza uralensis*	Root and rhizome	6.25
*Zingiber officinale*	Rhizome (undried)	6.25
*Zizyphus jujuba*	Fruit	6.25

**Table 3:** Primers used for qPCR.

Target Gene	Reference Sequence	Primer Sequence
TNF-α	NM_013693.3	F: 5′-TTCTGTCTACTGAACTTCGGGGTGATCGGTCC-3′
		R: 5′-GTATGAGATAGCAAATCGGCTGACGGTGTGGG-3′
IL-6	NM_031168.2	F: 5′-TCCAGTTGCCTTCTTGGGAC-3′
		R: 5′-GTGTAATTAAGCCTCCGACTTG-3′
IL-1β	NM_008361.4	F: 5′-ATGGCAACTGTTCCTGAACTCAACT-3′
		R: 5′-CAGGACAGGTATAGATTCTTTCCTTT-3′
MCP-1	NM_011333	F: 5′-GCTACAAGAGGATCACCAGCAG-3′
		R: 5′-GTCTGGACCCATTCCTTCTTGG-3′
IL-10	NM_010548	F: 5′-CGGGAAGACAATAACTGCACCC-3′
		R: 5′-CGGTTAGCAGTATGTTGTCCAGC-3′
iNOS	NM_010927.4	F: 5′-GGCAGCCTGTGAGACCTTTG-3′
		R: 5′-GCATTGGAAGTGAAGCGTTTC-3′
COX-2	NM_011198.4	F: 5′-TGAGTACCGCAAACGCTTCTC-3′
		R: 5′-TGGACGAGGTTTTTCCACCAG-3′
β-actin	NM_007393.5	F: 5′-AGAGGGAAATCGTGCGTGAC-3′
		R: 5′-CAATAGTGATGACCTGGCCGT-3′

F, forward; R, reverse.

**Table 4:** Primary and secondary antibodies used for Western blot analysis.

Antibody	Corporation	Product No.	RRID	Dilution Rate
P-ERK	Cell Signaling	#4377	AB_331775	1:1000
ERK	Cell Signaling	#9102	AB_330744	1:1000
P-p38	Cell Signaling	#9211	AB_331641	1:1000
P38	Cell Signaling	#9212	AB_330713	1:1000
P-JNK	Cell Signaling	#9251	AB_331659	1:1000
JNK	Cell Signaling	#9252	AB_2250373	1:1000
β-actin	Cell Signaling	#4970	AB_2223172	1:1000
P-IκBα	Cell Signaling	#2859	AB_561111	1:1000
IκBα	Cell Signaling	#4814	AB_390781	1:1000
P-NF-κB p65	Cell Signaling	#3033	AB_331284	1:1000
NF-κB p65	Cell Signaling	#8242	AB_10859369	1:1000
Lamin B1	Cell Signaling	#13435	AB_2737428	1:1000
iNOS	Cell Signaling	#13120	AB_2687529	1:1000
COX-2	Cell Signaling	#4842	AB_2085144	1:1000
2nd anti-mouse	Cell Signaling	#7076	AB_330924	1:5000
2nd anti-rabbit	Cell Signaling	#7074	AB_2099233	1:5000

## Data Availability

The original contributions presented in the study are included in the article; further inquiries can be directed to the corresponding authors.
